# Plasma cell-free DNA methylation marks for episodic memory impairment: a pilot twin study

**DOI:** 10.1038/s41598-020-71239-9

**Published:** 2020-08-25

**Authors:** M. Konki, N. Lindgren, M. Kyläniemi, R. Venho, E. Laajala, B. Ghimire, R. Lahesmaa, J. Kaprio, J. O. Rinne, R. J. Lund

**Affiliations:** 1 Turku Bioscience Centre, University of Turku and Åbo Akademi University, 20520 Turku, Finland; 2 Turku Doctoral Programme of Molecular Medicine, University of Turku, 20014 Turku, Finland; 3 Drug Research Doctoral Program, University of Turku, 20014 Turku, Finland; 4 Turku PET Centre, University of Turku, 20520 Turku, Finland; 5 Institute for Molecular Medicine Finland, University of Helsinki, 00014 Helsinki, Finland; 6 Department of Public Health, University of Helsinki, 00271 Helsinki, Finland

**Keywords:** DNA methylation, Dementia, Diagnostic markers, Neurodegenerative diseases, Methylation analysis

## Abstract

Decline in episodic memory performance usually causes the first clinical symptoms of Alzheimer’s disease. At present, Alzheimer’s disease can only be diagnosed at a very late stage when neurodegeneration and cognitive impairment is already irreversible. New early disease markers are needed for earlier and more efficient Alzheimer’s disease intervention. To identify early disease markers, we implemented a genome-wide bisulphite sequencing method for the analysis of plasma cell-free DNA methylation profiles and compared differences associated with episodic memory performance in Finnish twin pairs. A noticeable amount of cell-free DNA was present in plasma, however, the amounts as well as the genomic coverage of these fragments varied substantially between individuals. We found no significant markers associated with episodic memory performance in the twins’ plasma cell-free DNA methylation profiles. Furthermore, our results indicate that due to the low genomic coverage of cell-free DNA fragments and the variety in these fragments between individuals, the implemented genome-wide bisulphite sequencing method is not optimal for comparing cell-free DNA methylation differences between large groups of individuals.

## Introduction

Episodic memory (EM) is the memory type that starts to decline early in Alzheimer’s disease (AD) causing the usual first symptoms of dementia, like problems in remembering recent events and setting these events to the timeline. Alzheimer’s disease is the most common cause of dementia, inflicting tens of millions of patients worldwide^1^. Currently, AD can be clinically diagnosed only at a very late stage when neurodegeneration is far-progressed and patients’ cognitive impairment is already irreversible. While the initial triggers for AD are not completely known, the disease progression may start already 20–30 years before it can be diagnosed^2^. New early diagnostic or prognostic markers would be valuable enabling early intervention studies and evaluation of their effectiveness prior to the stage when severe neuronal damage has occurred in the disease process. Others and we have previously identified DNA methylation marks associated with AD or dementia in specific brain regions and in peripheral whole blood^3–5^. Plasma circulating cell-free DNA (cfDNA) methylation marks have emerged as a potential method for monitoring of cancers, rejection of organ transplantation and in prenatal diagnostics^6–8^. Whether these marks could be utilized to detect dementia is not clear. As cfDNA has been detected to leak into plasma from dying cells in cancers, cfDNA could be leaking to the blood circulation from the degenerating neuronal cells as well^9^. In this pilot study, our goal was to test and optimize the method for plasma cfDNA methylation sequencing and determine whether cfDNA methylation marks associated with EM performance can be detected in Finnish twin study participants.

## Results

### Optimization of the sample preparation method for cell-free DNA methylome analysis

To optimize the sample preparation method for cfDNA methylome analysis, we first examined the impact of anticoagulant type on the cfDNA yields. For this purpose, whole blood was collected into EDTA (n = 3) and CPT (n = 4) tubes. Plasma was fractionated and cfDNAs were isolated from each sample by using 1,000 μl volume of plasma. The cfDNA yields varied between 2.05 and 17.08 ng. No statistically significant differences were detected in the cfDNA yields between EDTA or CPT plasma (paired t-test p-value 0.20, Supplementary Fig. S1). We then examined impact of plasma volume on the cfDNA yields. For this purpose, cfDNAs were isolated from 1,000, 750 and 500 μl of EDTA plasma with Qiagen QIAamp Circulating Nucleic Acid kit that is optimized for up to 5 ml plasma volumes. When using 500 μl of EDTA plasma as a starting volume, two of three replicates did not yield any cfDNA indicating that this starting volume is not robust for cfDNA isolation. When using 750 and 1,000 ul of EDTA plasma cfDNAs were successfully isolated from all samples. There were no differences (t-test p-value > 0.20) in the cfDNA yields between different starting volumes of plasma (Supplementary Fig. S1), although, there was more variation in the isolated cfDNA yields with the lower plasma volumes. Our results indicated that further increase in the plasma volume would not increase the yield.

Next, we determined whether bisulphite sequencing libraries can be prepared from these samples and whether the type of the anticoagulant and starting amount of cfDNA affect the number of detected CpG sites. Library preparation from all the samples was successful. The libraries were prepared from 2.0–10 ng of cfDNA isolated from EDTA plasma and 1.0–17 ng of cDNA isolated from CPT plasma. According to the results, there were no differences in the number of CpG sites detected in samples prepared from EDTA plasma when compared to the CPT plasma (t-test p-value 0.7). Furthermore, the correlation between the number of detected CpG sites and the amount of cfDNA used as starting material for library preparation was not statistically significant (Pearson’s correlation 0.28, p-value 0.44, Supplementary Fig. S2).

### Pilot analysis of plasma cfDNA markers associated with episodic memory performance in Finnish twin pairs

To identify plasma cell-free markers for EM performance, we then repeated the analysis by using samples collected from Finnish twin cohort. Our first null hypothesis was that the plasma cell-free DNA yields do not correlate with the EM performance. To test this hypothesis, cell-free DNAs were isolated from 1 ml of plasma collected from seven Finnish twin pairs (see materials and methods section for details). Of these twin pairs four were discordant for EM impairment, two pairs were cognitively preserved and in one pair both siblings were affected (Table 1). The cfDNA yields from the 14 individuals varied between 3.9 and 30.90 ng, however, did not correlate with the Logical Memory delayed recall (r = − 0.16, p = 0.58) or delayed word list recall (r = − 0.17, p = 0.57) scores. Therefore, the null hypothesis was not rejected.

**Table 1:** Description of the seven Finnish twin pairs included in the plasma cell-free DNA methylation analysis.

Twin pair ID	Zygosity	Gender	EM status	EM z-score	*APOE* genotype	Amount of DNA isolated (ng)
MZ1	1	Male	Impaired	− 1.75	33	4.9
MZ1	1	Male	Impaired	− 2.67	33	7.5
MZ2	1	Male	Normal	1.23	33	10.7
MZ2	1	Male	Normal	− 0.11	33	11.6
DZ1	2	Female	Impaired	− 1.19	33	30.9
DZ1	2	Female	Normal	0.56	33	6.8
DZ2	2	Male	Normal	− 0.52	33	8.7
DZ2	2	Male	Normal	− 0.52	33	5.6
DZ3	2	Male	Normal	0.30	34	5.5
DZ3	2	Male	Impaired	− 3.08	34	19.5
DZ4	2	Female	Normal	0.59	33	11.4
DZ4	2	Female	Impaired	− 2.80	34	8.0
DZ5	2	Female	Impaired	− 2.13	44	3.9
DZ5	2	Female	Normal	1.12	34	5.4

Their mean age was 74.1 years (range 73–76).

Zygosity (1: monozygotic, 2: dizygotic), *EM* episodic memory.

**Figure 1:**
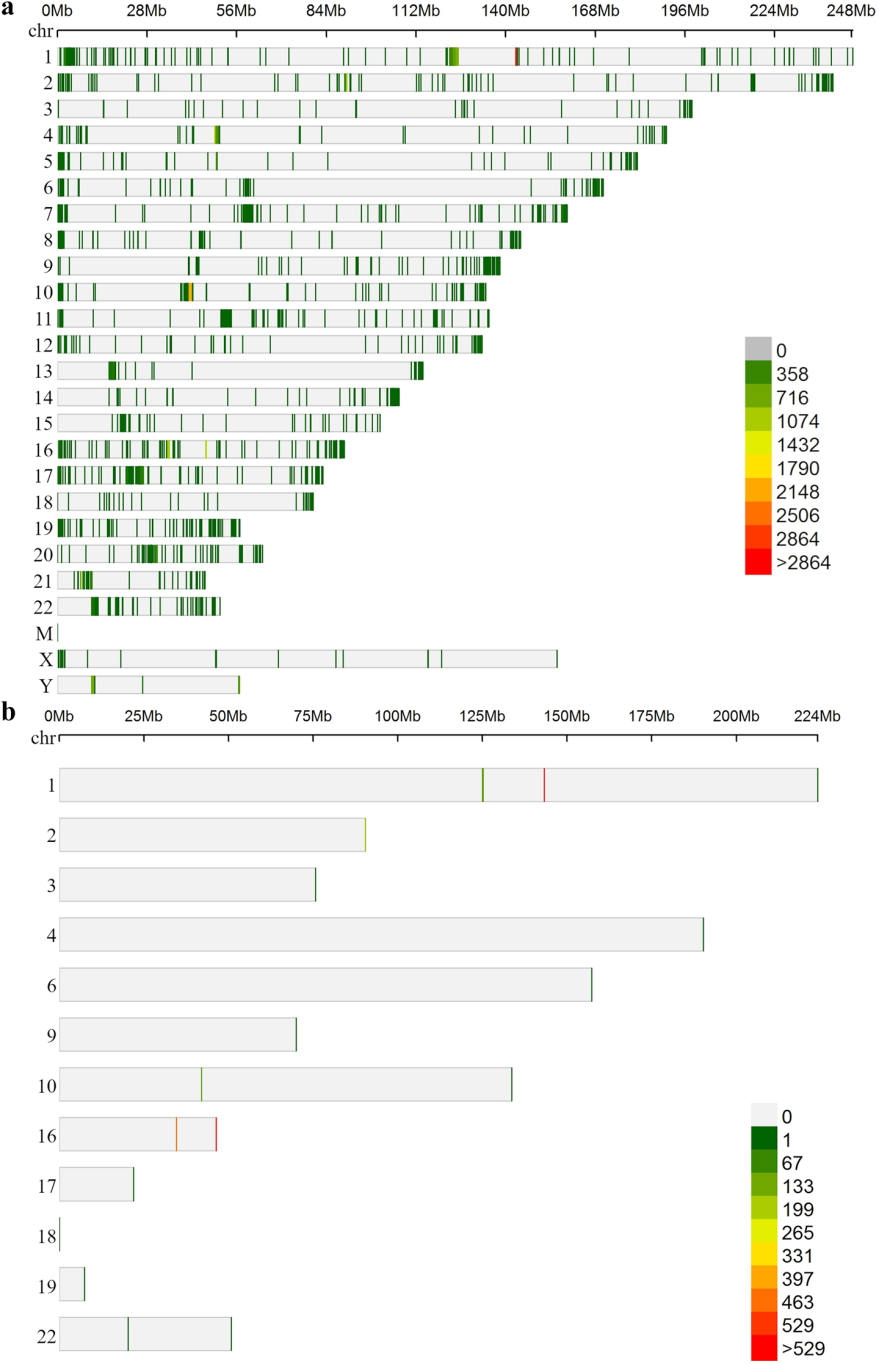
CpG sites detected with plasma cell-free DNA bisulphite sequencing. (**a**) CpG sites detected with ≥ 10 × sequencing coverage in one or more twin study participants. (**b**) CpG sites detected with ≥ 10 × sequencing coverage in all of the twin participants. The CpG sites in figure (**b**) were included in the differential methylation analysis. Color scaling: number of sites detected within one million base pair windows.

**Figure 2:**
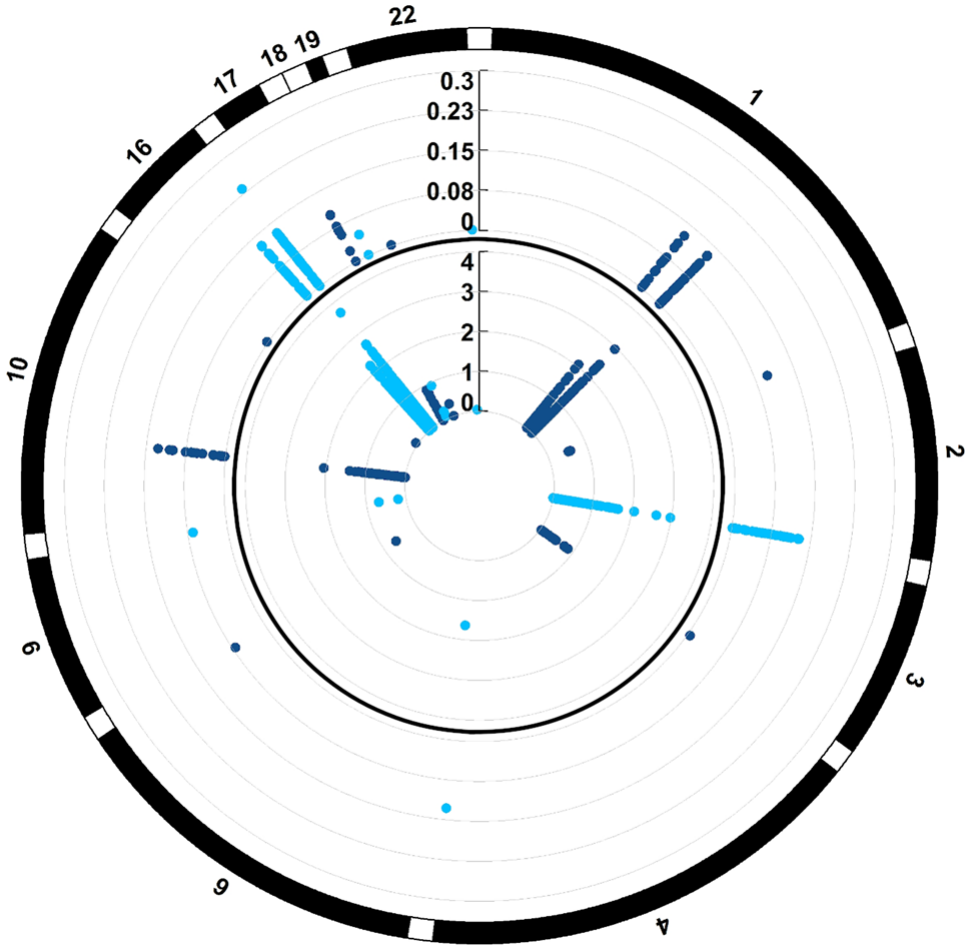
Methylation differences associated with episodic memory performance in the Finnish twins. The manhattan plots show multiple test corrected log10(p)-values in the outer circle and unadjusted log10(p)-values in the inner circle for CpG sites that were included in the analysis. Generalised linear mixed effects model (PQLseq) was utilised to test for plasma cfDNA methylation marks associated with EM performance within the seven twin pair sample set.

Our second null hypothesis was that cell-free DNA methylation marks associated with EM performance cannot be detected in plasma. To test this hypothesis, we characterised plasma cfDNA methylomes of the seven twin pairs (14 individuals) with whole genome bisulphite sequencing and analysed whether plasma cfDNA methylation is associated with EM performance. The number of CpG sites detected in each individual with 10 × sequencing coverage varied between 4,023 and 13,414. A total number of 2,089 CpG sites were detected in all 14 individuals with 10 × sequencing coverage and were included in the differential methylation analysis (Fig. 1). Of these sites 99.52% were located in intergenic regions of the genome. Differential methylation was analysed using a binomial mixed model implemented in R package PQLseq^10^. The EM z-score was set as the test variable, gender, age, zygosity and *APOE* genotype were included as fixed effects and twin pair ID was included as random effect in the model. *APOE* genotype was added as a fixed effect variable since it is a significant risk factor for AD and our previous results indicate that it can correlate with DNA methylation in peripheral blood^3^. The model converged in 2002 of the 2089 CpG sites. To control for multiple testing, p-values were adjusted by combining the significance levels of nearby CpG sites, using RADMeth^11^, and corrected using FDR. After correction, we detected no significant methylation differences associated with EM performance (adjusted FDR < 0.05) (Fig. 2, Supplementary Table S1). Therefore, we did not reject the null hypothesis.

As a conclusion, no differentially methylated sites associated with EM performance were detected in plasma circulating cfDNA with our sample size and analysis methods.

## Discussion

In this study, we implemented a method for whole genome bisulphite sequencing of plasma cfDNA. The method was piloted by comparing plasma cfDNA methylation differences associated with EM performance in Finnish twin pairs. To our knowledge, there is no previous published studies on genome-wide characterisation of plasma cfDNA methylation markers for EM impairment even though several groups have identified DNA methylation differences associated with neurodegenerative diseases in brain tissue and peripheral blood^3–5^. However, plasma cell-free DNA methylation markers have been characterized for other human diseases, like cancers, and biological conditions, like prenatal diagnostics and organ transplant monitoring using similar sequencing-based methods as well as methylation arrays^6–8^. In addition, a recent study that utilised targeted bisulphite pyrosequencing reported neuronal tissue specific methylation pattern in LIM homeobox 2 (*LHX2*) gene in plasma cfDNA associated with AD^12^.

The cfDNA amounts, isolated from 1 ml twin plasma samples varied from less than four nanograms to more than thirty nanograms. Our results suggest that there is noticeable amount of cfDNA circulating in peripheral blood, however, the amount may vary a lot between individuals. Furthermore, plasma cfDNA amounts did not correlate with EM performance, in contrast to the findings Pai and his group discovered in their study^12^. However, Pai’s group quantified circulating cfDNA in AD patients who have more severe neurodegeneration and cognitive impairment than our study participants. Thus, it is possible that plasma cfDNA levels increase as neurodegeneration progresses further. We detected between 4,023 and 13,414 CpG sites with 10 × sequencing coverage in each twin sample, which indicates that also the genomic coverage of the cfDNA fragments varies substantially between individuals and therefore the method is not optimal to compare large groups of individuals. A vast majority of the detected sites located in the intergenic regions of the genome, especially in centromeres and telomeres, which suggests that these functional genomic regions are highly represented in plasma cfDNA. We did not detect any significant differences in methylation of the cfDNA fragments associated with EM impairment. Since our study material consisted of samples from individuals with relatively mild cognitive deficits, it is possible that DNA methylation markers would be detectable at a later stage when neurodegeneration is more severely progressed.

The low number of twin pairs that we were able to obtain for this study was a limitation in the pilot analysis of plasma cell-free DNA methylation differences associated with EM performance. However, we detected no promising marker candidates and our results indicate that both cfDNA amounts and genomic coverage of these fragments vary remarkably between individuals. After all, only 2,089 of the 28 million CpG sites in human genome were detected in all of our study participants with sufficient sequencing coverage. Thus, we think that no reliable methylation marks associated with mild EM impairment can be identified in plasma cfDNA with the implemented genome-wide bisulphite sequencing method that we applied even if the number of twin pairs could be increased.

## Materials and methods

### Description of the study participants

A total number of 559 same-sex twin pairs (born 1938–1944) from the older Finnish Twin Cohort participated in a telephone-based cognitive screening. According to primary information about their cognitive performance 45 individuals; nine monozygotic twin pairs and nine dizygotic twin pairs as well as eight non-twin controls, were invited for further assessment at the Turku PET Centre, Finland, where they underwent blood sampling and neurophysiological testing in 2014–2017^13–15^. From the 18 twin pairs we selected the seven first ones with plasma sample available at the time of the study; two monozygotic and five dizygotic twin pairs, who were examined at PET Centre before July 2016, for a cell-free DNA methylation characterization pilot (Table 1). The participants were administered a neuropsychological test battery that included the delayed word list recall from the Consortium to Establish a Registry for Alzheimer’s disease Neuropsychological Battery (CERAD-NB) and Logical Memory delayed recall from the Wechsler Memory Scale-Revised (WMS-R). The test performances were transformed into standard deviation (SD) units based on age-appropriate Finnish norms^16,17^. Following the Jak/Bondi neuropsychological criteria, participants were defined to have EM impairment if they had ≤ − 1 SD or poorer performance in both delayed verbal recall tests^18^. According to these criteria, four twin pairs were discordant for EM impairment (Table 1). The EM z-score values in Table 1 are the combined means from CERAD-NB and WMS-R test scores. In addition to the twin samples, we collected test plasma samples into EDTA and CPT tubes from eight non-related controls in total for testing and optimizing the sample preparation protocol. (Supplementary Table S2).

The study was approved by the Ethics Committee of Hospital District of Southwest Finland. The study was conducted according to good clinical and scientific practices and following the ethical principles of the Declaration of Helsinki. Informed consent was obtained from all study participants.

### DNA isolation

DNA was isolated from plasma with Qiagen’s QIAamp Circulating Nucleic Acids kit. For library preparation testing DNA was isolated from 500 µl, 750 µl or 1,000 µl of plasma. Plasma was extracted from EDTA or CPT blood samples within two hours after sampling, snap-frozen in dry ice and stored in −80 °C before DNA isolation. Twin samples were stored in CPT tubes and 1,000 µl of plasma was used in DNA isolation per sample. Isolated DNA quality was analysed with Agilent Bioanalyzer 2100 High-Sensitivity DNA assay and DNA was quantified with Invitrogen Qubit 2.0 dsDNA HS assay.

### Library preparation

Bisulphite sequencing library preparation test was started with 2 to 10 ng of plasma DNA per sample. Library preparation conditions for the test samples can be seen in more detail in Supplementary Table S2. Library preparation started with end-filling/A-tailing reaction with Klenow fragment (New England Biolabs) and nucleotide mix containing 10 × concentration of ATP compared to other nucleotides, and unmethylated lambda DNA. The reaction mix was incubated 30 min in 30 °C and 30 min in 37 °C. In the next step Illumina TruSeq sequencing adapters were ligated to the DNA fragments with T4 DNA ligase (New England Biolabs) and overnight incubation in 16 °C. After adapter ligation, libraries were purified with AMPure XP beads in 2.5X bead concentration. Bisulphite conversion was carried out with Invitrogen MethylCode Bisulfite Conversion kit, according to the manufacturer’s instructions. 25 ng of carrier DNA (sheared *E. Coli* DNA) was added to each library sample before conversion. Libraries were amplified in an 18 cycle PCR and purified two times with AMPure XP beads, 1.2X and 1.5X bead concentration. Library concentrations were measured with Qubit 2.0 HS DNA assay and quality analysed with Bioanalyzer 2,100 High Sensitivity DNA assay. The twin sample bisulphite sequencing libraries were prepared from 3 ng of plasma DNA.

### Sequencing and data analysis

Libraries were sequenced with Illumina HiSeq 2500/3000 1 × 50 bp chemistry. The trimmed reads (Trim Galore 0.4.1^19^) were mapped to hg38 reference genome with Bismark 0.14.5^20^. Centromere regions, haplotype and sex chromosomes and repetitive regions that had very high sequencing coverage were filtered out from the data set. Differential methylation was tested at individual CpG sites with PQLseq^10^ (version 1.1) software. PQLseq was utilised to identify plasma cfDNA methylation markers associated with EM performance in the whole seven twin pair sample set. PQLseq implements a generalised linear mixed effects model to test for differential methylation associated with a given trait. All seven pairs (14 twins) were included in the mixed model analysis, however, only CpG sites that were detected with at least 10 × sequencing coverage in all individuals were included. 10 × sequencing coverage means that a CpG site is covered by ten unique sequencing reads after mapping, i.e. the site is sequenced ten times in the library sample. EM z-score was set as the predictor variable. Gender, age, twin zygosity and *APOE* genotype were included as fixed effects and twin pair ID as a random effect in the model. The p-values obtained from PQLseq analysis were adjusted by combining the p-values of nearby CpG sites and FDR corrected for multiple testing using methPipe/RADMeth (version 3.4.3)^11^. The threshold for differential methylation was adjusted FDR < 0.05.

## Supplementary information

Supplementary file1

Supplementary file2

## Data Availability

All the data supporting the findings in this study are available within the article or upon request from the corresponding author.
