# Supplementary material for: Plasma cell-free DNA methylation marks for episodic memory impairment: a pilot twin study
Source: Sci Rep. 2020 Aug 25;10:14192. doi: 10.1038/s41598-020-71239-9 (PMC7447764; doi:10.1038/s41598-020-71239-9)
Supplement: Supplementary file 1 — Supplementary file1 [file 41598_2020_71239_MOESM1_ESM.pdf]

Plasma cell-free DNA methylation marks for episodic memory impairment: a pilot twin study

Konki, M.[1,2*], Lindgren, N.[3,4], Kyläniemi, M.[1], Venho, R.[1], Laajala, E.[1], Ghimire, B.[5], Lahesmaa, R.[1], Kaprio, J.[5,6], Rinne, J. O.[4], Lund, R. J.[1]

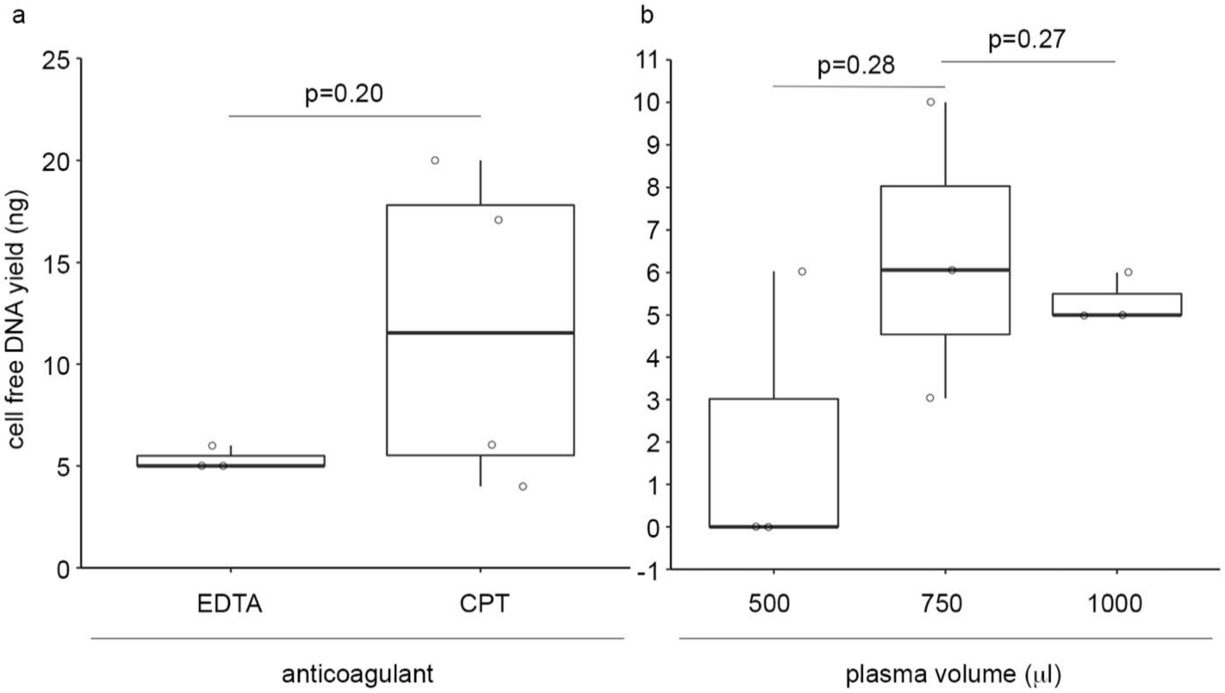

Supplementary Figure S1. Cell-free DNA yields. a) Cell-free DNAs were isolated from 1 ml of EDTA (n=3) and CPT plasma (n=4). The concentrations were measured and difference were compared with Student's t-test. b) Cell-free DNAs were isolated from different volumes of EDTA plasma as indicated in the figure. The differences in the yields were compared with paired Student's t-test.

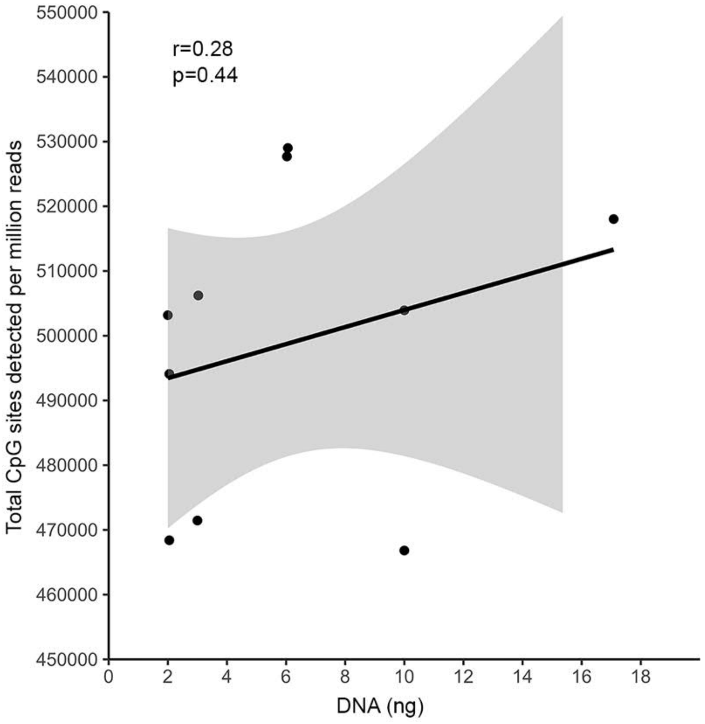

Supplementary Figure S2. Correlation between the amount of plasma DNA used in library preparation and the number of CpG sites detected per 10^6 uniquely aligned reads (Pearson correlation 0.28, p=0.44).

Supplementary table S1. Plasma cell-free DNA differential methylation analysis results.

Supplementary table S2. Description of the test samples used in optimizing of the plasma DNA isolation and bisulphite sequencing library preparation protocols.

ID	Participant ID	Blood tube type	Plasma amount used in DNA isolation (µl)	Total amount of isolated DNA (ng)	DNA amount used for library preparation (ng)	Sequences analysed in total	Number of uniquely aligned reads	Total number of CpG sites detected
1	1	EDTA	1000	5	2.5	10478393	7391368	3652099
2	1	EDTA	750	6.6	6.6	11751634	8080343	4274608
3	1	EDTA	500	6.3	6.3			
4	2	EDTA	1000	5	2.5	8458538	5899375	2763253
5	2	EDTA	750	10	10.0	9920860	6851543	3452704
6	3	EDTA	1000	6	3.0	10275044	7388356	3483232
7	3	EDTA	750	3.3	3.3	12725950	9005590	4558839
8	4	CPT	1000	6.3	6.3	3910474	2763957	1458532
9	5	CPT	1000	4	2.0	12335475	8770715	4413243
11	6	CPT	1000	17.8	17.8	4577127	3180919	1647809
13	8	CPT	1000	20	10.0	14573949	10268334	4793427
14	8	CPT	1000	3.2	1.6	15441075	10677666	5165726
